# Differential Microbial Communities of Omnivorous and Herbivorous Cattle in Southern China

**DOI:** 10.1016/j.csbj.2018.02.004

**Published:** 2018-02-15

**Authors:** Susanna K.P. Lau, Jade L.L. Teng, Tsz Ho Chiu, Elaine Chan, Alan K.L. Tsang, Gianni Panagiotou, Shao-Lun Zhai, Patrick C.Y. Woo

**Affiliations:** a State Key Laboratory of Emerging Infectious Diseases, Hong Kong, Hong Kong; b Department of Microbiology, Li Ka Shing Faculty of Medicine, The University of Hong Kong, Hong Kong, Hong Kong; c Research Centre of Infection and Immunology, The University of Hong Kong, Hong Kong, Hong Kong; d Carol Yu Centre for Infection, The University of Hong Kong, Hong Kong, Hong Kong; e Collaborative Innovation Center for Diagnosis and Treatment of Infectious Diseases, The University of Hong Kong, Hong Kong, Hong Kong; f Systems Biology and Bioinformatics Group, School of Biological Sciences, Faculty of Sciences, The University of Hong Kong, Hong Kong, Hong Kong; g Systems Biology and Bioinformatics, Leibniz Institute for Natural Product Research and Infection Biology, Hans Knöll Institute, Jena, Germany; h Guangdong Key Laboratory of Animal Disease Prevention, Animal Disease Diagnostic Center, Institute of Animal Health, Guangdong Academy of Agricultural Sciences, Guangzhou, China

**Keywords:** Cattle, Microbiome, Diet, Omnivore, Microbial diversity

## Abstract

In Hong Kong, cattle were traditionally raised by farmers as draft animals to plough rice fields. Due to urbanization in the 20th century, they were gradually abandoned and became wild cattle straying in suburban Hong Kong. Recently, these cattle were observed to have become omnivorous by eating leftover barbeque food waste in country parks. Microbiome analysis was performed on fecal samples of the omnivorous cattle using deep sequencing and the resulting microbiome was compared with that of traditional herbivorous cattle in Southern China. A more diverse gut microbiome was observed in the omnivorous cattle, suggesting that microbiota diversity increases as diet variation increases. At the genus level, the relative abundance of *Anaeroplasma*, *Anaerovorax*, *Bacillus*, *Coprobacillus* and *Solibacillus* significantly increased and those of *Anaerofustis*, *Butyricimonas*, *Campylobacter*, *Coprococcus*, *Dehalobacterium*, *Phascolarctobacterium*, *rc4.4*, RFN20, *Succinivibrio* and *Turicibacter* significantly decreased in the omnivorous group. The increase in microbial community levels of *Bacillus* and *Anaerovorax* likely attributes to the inclusion of meat in the diet; while the decrease in relative abundance of *Coprococcus*, *Butyricimonas*, *Succinivibrio*, *Campylobacter* and *Phascolarctobacterium* may reflect the reduction in grass intake. Furthermore, an increased consumption of resistant starch likely resulted in the increase in abundance of *Anaeroplasma*. In conclusion, a significant change in the gut microbial community was observed in the omnivorous cattle, suggesting that diet may be one of the factors that may signal an adaptation response by the cattle to maintain feed efficiency as a consequence of the change in environment.

## 1 Introduction

Cattle, collectively classified as *Bos taurus*, are the most common group of large domesticated ungulates and the most widespread species of the genus *Bos*. They are raised for meat, milk and other dairy products, as well as for pulling carts and plowing. As ruminants, they have four-chambered stomachs for the efficient breakdown of indigestible plant material via fermentation and this process is performed by a complex microbiota in the rumen that converts food into energy. Hence, these gut microorganisms have an important role in maintaining the physiology of the host.

In the literature, microbiome projects on cattle have mainly focused on determining the effects of different dietary treatments on gut bacterial composition. This includes varying concentrations of fiber and starch [1,2] to the addition of supplements such as copper, zinc, manganese [3] and mineral salts [4]. These studies are beneficial in exploring approaches that may improve milk yield, meat mass, productivity and metabolic activity of cattle, especially for the dairy cow. On the other hand, some studies characterize differences in fecal microbiota due to the presence of pathogen [5] and disease [6] and the effect of potential treatment for metabolic disorders [7]. Finally, there are studies which examine the gut microbiota of cattle in their natural status without additional treatment or stimulus, such as during lactation [8] or comparing free-grazing individuals within or across communities [9,10]. These reports allow us to understand the ways in which the intestinal microbiome interact and contribute to the wellbeing of the animal and how changes in the environment or conditions may impact their gut microbial ecosystem.

In Hong Kong, brown cattle were traditionally raised by local farmers for centuries as draft animals to plough rice fields. When the population gradually urbanized in the last few decades of the 20th century, the cattle were abandoned. Their descendants became wild cattle straying in the suburban areas of Hong Kong, such as the country parks. In recent years, these cattle surprisingly ate the food waste left by country park visitors at the barbeque sites and became omnivorous. Due to the change in diet, we hypothesized that the microbiomes of these omnivorous cattle may adapt to the new diet. As microbiome communities are vital for the breakdown and absorption of nutrients, which contributes to the health and well-being of the cattle, it is important to determine the effect of the dietary change on the gut microbiota. In this study, we characterized and compared the microbiome of omnivorous cattle in Hong Kong with that of the traditional herbivorous cattle, and explored the hypothesis that a change in diet from herbivorous to omnivorous significantly impacts the microbiota composition.

## 2 Materials and Methods

All animal experimental procedures were performed under protocols approved by the University of Hong Kong Committee on the Use of Live Animals in Teaching and Research (CULATR 3330-14).

### 2.1 Collection of Cattle Fecal Samples

**Fig. 1:**
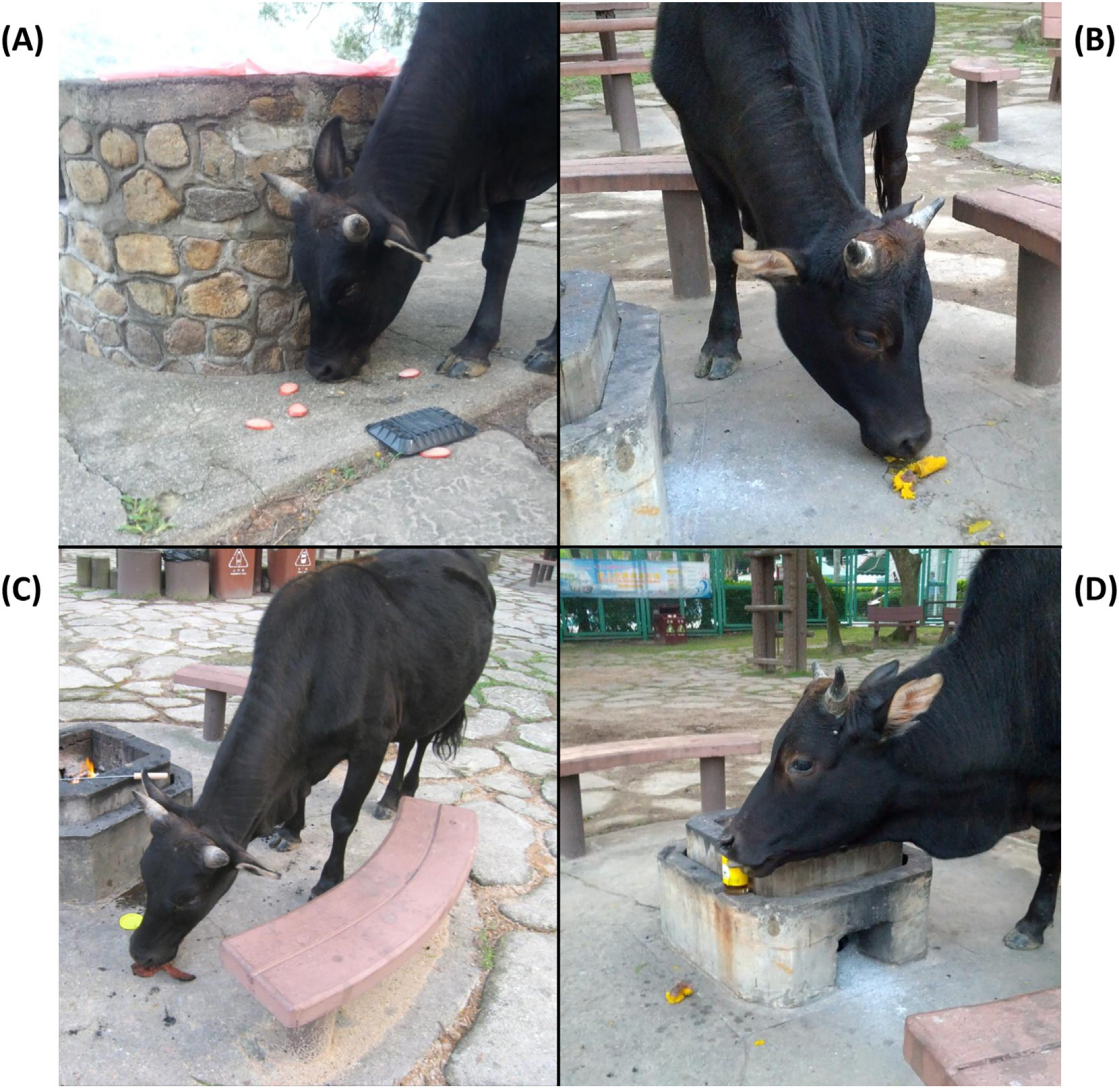
Photos of omnivorous cattle consuming food waste from a barbeque site at Sai Kung Country Park. (A) Cow eating raw sausages, (B) cow eating leftover cooked sweet potato, (C) cow eating a piece of beef steak, and (D) cow licking honey from the jar.

Ten fecal samples (one from each cow) were collected from healthy wild cattle located at the Sai Kung Country Park in Hong Kong that consumed an omnivorous mixed diet of grass and barbeque food waste (Group M; mixed) (Table 1). The Sai Kung Country Park is a 3000 ha wild country park in the western part of Sai Kung Peninsula, Hong Kong [11]. The park is a mountainous terrain popular for hiking and has several trailside barbeque sites and picnic areas located within. The cattle roam these barbeque sites and feed on food waste left in rubbish bins or given to them by barbeque parties, thus their food consumption includes a mixed diet of grass and barbeque food waste including raw or partially cooked meat, such as beef, pork, chicken, or fish, as well as sweet potato, honey, corn and bread (Fig. 1). Another ten fecal samples (one from each cow) were collected from healthy cattle resided on a hill in a free-grazing farm located in Guangzhou, China, that consumed a main diet of grass and plants (Group G; grass) and served as the control herbivore group for microbiome comparison (Table 1). Fecal samples from cattle were collected immediately after natural defecation, stored immediately on ice, then transported to the laboratory and frozen at −80 °C prior to analyses. All samples were obtained from the inside of the feces using sterilized equipment, with no contact with soil or other pollution sources. PCR amplification and sequencing of the mitochondrial cytochrome-b gene was performed to validate the twenty cattle were of the species *Bos taurus* (data not shown).

**Table 1:** Characteristics and diet of cattle sampled in this study.

	Dietary group^a^	
	Mixed	Grass
Number of cattle sampled	10	10
Breed	*Bos taurus*	*Bos taurus*
Age	Adult	Adult
Status	Healthy	Healthy
Location	Sai Kung Country Park, Hong Kong	Free-grazing farm, Guangzhou, China
Diet composition	Forage and barbeque food waste (omnivorous)	Forage only (herbivorous)
Number of sample taken	1 fecal sample per cow	1 fecal sample per cow

a Mixed, Group M; grass, Group G.

### 2.2 DNA Extraction, PCR Amplification, and MiSeq Sequencing

Total genomic DNA was extracted from 200 mg of fecal sample using a QIAamp DNA Stool Mini Kit (Qiagen) according to the manufacturer's instructions. The genomic DNA and its quality were quantified and checked using Nanodrop spectrophotometer (ND1000; Thermo Fisher Scientific, Wilmington, DE, USA) and agarose gel electrophoresis, respectively. Primers that span the hypervariable regions V3–V4 of the bacterial 16S rRNA gene (Forward: 5′-CCTACGGGNGGCWGCAG-3′, Reverse: 5′-GGACTACHVGGGTAATCC-3′) were used for amplicon generation and sequencing using the MiSeq PE300 platform (Illumina) according to the manufacturer's instructions. Library preparation and sequencing were performed at the Centre for Genomic Sciences, The University of Hong Kong. Sequencing coverage was approximately 212,683 sequences per sample on average. Reads were submitted to the NCBI short-read archive (BioProject PRJNA371636 – Biosample accession numbers for individual animal samples sequencing data are SAMN06310326, SAMN06310355, SAMN06310356, SAMN06310361, SAMN06310362, SAMN06310375, SAMN06310392, SAMN06310393, SAMN06310399, SAMN06310428, SAMN06310429, and SAMN06310448–SAMN06310456).

### 2.3 Data Analysis

Raw sequence read data were assembled by fastq-join from ea-utils.1.1.2-537 [12] with all the unjoined reads filtered out. The joined paired-end reads were analyzed using the QIIME 1.8 pipeline [13] with default parameters. Operational taxonomic units (OTUs) were picked from the assembly paired-end reads via UCLUST [14] at 97% similarity, with OTUs fewer than 10 reads removed to avoid PCR sequencing errors, and representative sequences were selected from each OTU. Taxonomic assignments of OTUs were determined using UCLUST based on 16S rRNA gene reference sequences from GreenGenes taxonomy database (release 13_5) [15]. Alpha diversity of samples was calculated using a rarefaction curve from QIIME's alpha diversity pipeline [13]. Samples were rarefied to 124,000 reads (the least number of reads per sample), and diversity was calculated by the number of species per sequencing depth, Shannon index (estimation of the total diversity with both species richness and evenness taking into consideration) and Chao1 index (estimation of total species richness). Differences in the number of OTUs among the two dietary regimes were evaluated using an ANOVA. For multivariate analysis, Calypso [16] was used for the Statistical and Principle Components Analysis (PCA).

## 3 Results

### 3.1 Illumina Sequences

Raw reads were generated by Illumina MiSeq PE300 sequencing of the 20 fecal samples. 4,253,662 high quality joined reads were then obtained from the raw reads for downstream analyses via quality trimming, pair-end joining, and chimeric filtering. Individual samples that passed quality checking generated an average of 212,683 reads per sample. After clustering and taxonomic assignment at 97% similarity, OTUs with <10 observation counts were discarded, 68,181 unique OTUs were identified and the number of total OTUs for each individual sample ranged from 3955 to 12,251, with an average of 7983.

### 3.2 Composition of Bacterial Community at Phylum and Genus Level

At 97% similarity, alignments and phylogenetic assignments resulted in the identification of 26 phyla, 52 classes, 95 orders, 181 families, and 374 genera across the two bacterial domains.

**Fig. 2:**
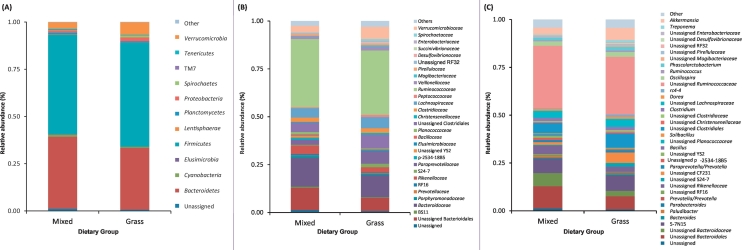
Relative abundance of bacteria composition across the two dietary groups at (A) phylum, (B) family and (C) genus levels. “Others” includes all phyla or genera with <0.1% relative abundance. Mixed, Group M; grass, Group G.

At the phylum level, >90% of the bacterial sequences were assigned to *Firmicutes*, *Bacteroidetes*, *Verrucomicrobia* and *Proteobacteria*. The relative abundance of *Bacteroidetes*, *Cyanobacteria* and *Tenericutes* were more dominant in the gut microbiota of Group M; while the abundance of *Verrucomicrobia* and *Proteobacteria* were more enriched in the gut microbiota of Group G (Fig. 2A).

At the family level, the most predominant family (>1% overall abundance across the two dietary groups) included *Ruminococcaceae*, *Bacteroidaceae*, *Paraprevotellaceae*, *Lachnospiraceae*, *Verrucomicrobiaceae*, *Veillonellaceae*, *Rikenellaceae* and *Clostridiaceae*, as well as those unclassified derived from *Bacteroidales* (order) and *Clostridiales* (order) (Fig. 2B). The relative abundance of the family *Bacteroidaceae*, *Rikenellaceae*, *Ruminococcaceae* and the unclassified *Bacteroidales* were more enriched in the gut microbiota of Group M; while *Paraprevotellaceae*, *Clostridiaceae*, *Lachnospiraceae*, *Veillonellaceae*, *Verrucomicrobiaceae* and the unclassified *Clostridiales* were predominant in the gut microbiota of Group G.

At the genus level, the most predominant genera included 5-7N15, *Akkermansia*, *Oscillospira*, *Phascolarctobacterium*, *Clostridium*, *Prevotella* and *Dorea*, as well as those unclassified derived from *Ruminococcaceae* (family), *Bacteroidales* (order), *Clostridiales* (order), *Bacteroidaceae* (family), *Lachnospiraceae* (family) and *Rikenellaceae* (family) (Fig. 2C). The relative abundance of the unclassified *Rikenellaceae*, unclassified *Bacteroidales* and unclassified *Bacteroidaceae* were more enriched in the gut microbiota of Group M; while the relative abundance of the genera *Akkermansia*, *Treponema*, *Phascolarctobacterium*, *rc4-4*, the unclassified CF231 and the unclassified S24-7 were predominant in the gut microbiota of Group G.

### 3.3 Comparison of Bacterial Diversity

**Fig. 3:**
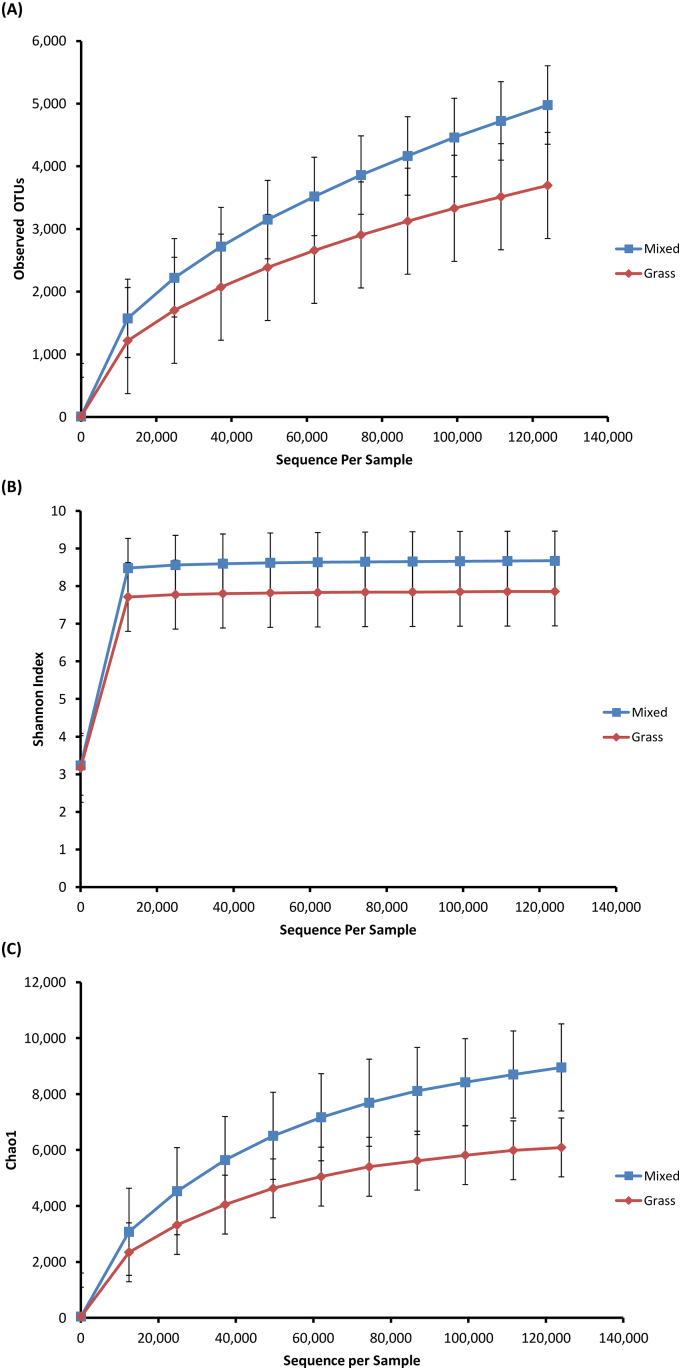
Alpha diversity of fecal samples across the two dietary groups represented by relative changes of rarefaction depth in terms of (A) number of observed OTUs, (B) Shannon index (diversity) and (C) Chao1 (richness). Mixed, blue square, Group M; grass, red diamond, Group G.

**Fig. 4:**
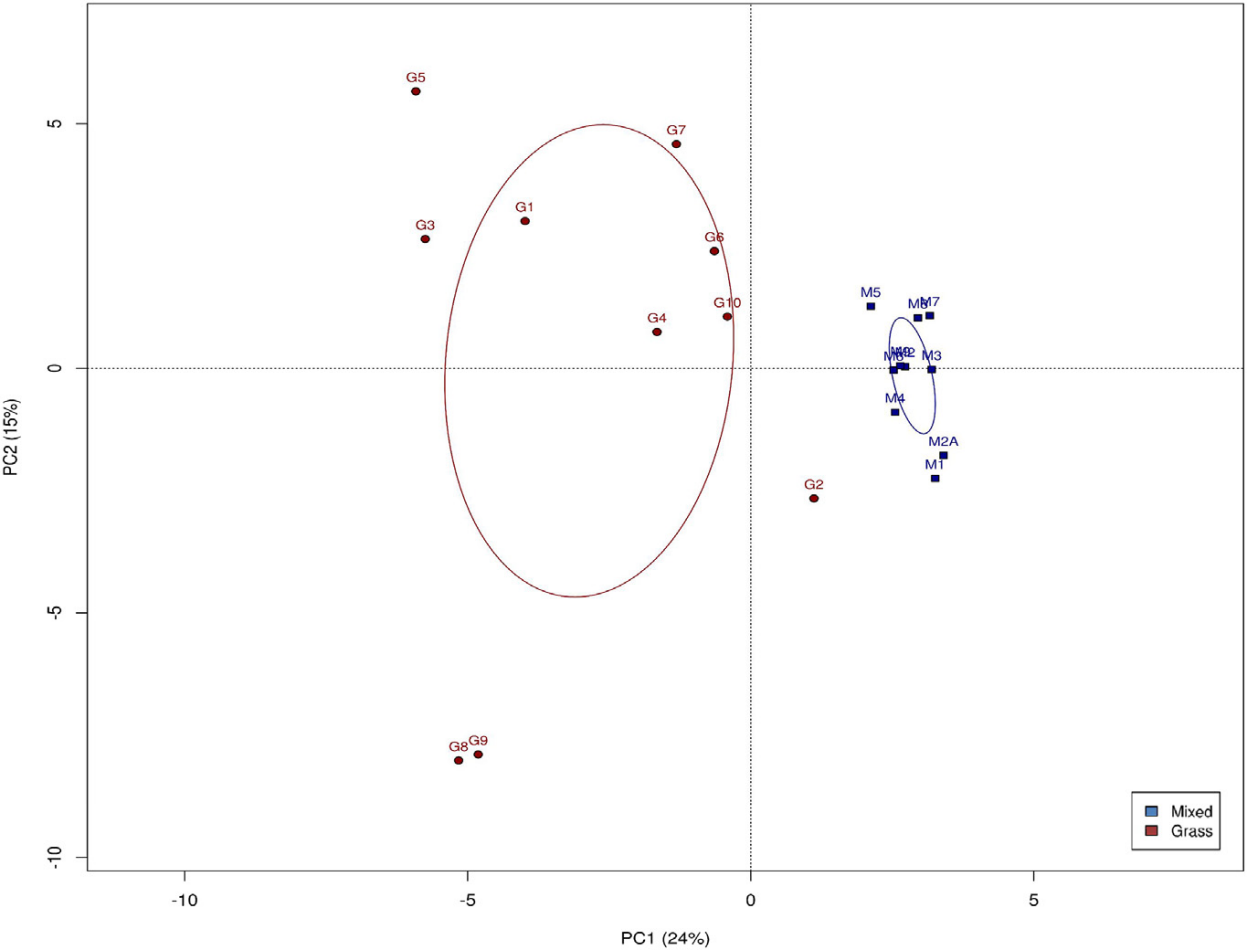
Principal component analysis (PCA) of fecal samples across the two dietary groups based on community structure in terms of the number of OTUs present. Each symbol represented one gut microbiota. Confidence intervals for eclipses around group centroids are 95%. Mixed, blue squares, Group M; grass, red circles, Group G.

In order to give an unbiased comparison of impacts on the alpha diversity due to the different dietary consumption, the OTU table was rarefied to the number of reads of the sample with the lowest number of reads, which is 124,000 in such case. At the maximum sub-sample depth, Group M samples were found to consist of around 1282 more observed OTUs than Group G samples (Fig. 3A). Consistent differences in Shannon diversity index (evenness) and Chao1 values (richness) were also observed across the two sample groups at sub-sample depth point. The bacterial communities present in the microbiota of Group M (H = 8.674) had a higher Shannon index than that of Group G (H = 7.860), meaning an increase in OTU number as well as evenness of the distribution of individuals among the OTUs (Fig. 3B). Group M (*x* = 0.991) also had a higher Chao1 value than Group G (*x* = 0.981), indicating higher richness, i.e., increase in OTU counts per sample, in Group M (Fig. 3C). PCA was used to demonstrate the varieties of community structure of individual samples from the two dietary groups. Samples were found to be clustered together according to their dietary groups and a similar pattern was observed across PCA at all levels. Fig. 4 represent these clusters at the level of OTUs present.

### 3.4 Comparison of Bacteria Profiles at the Genus Level

**Fig. 5:**
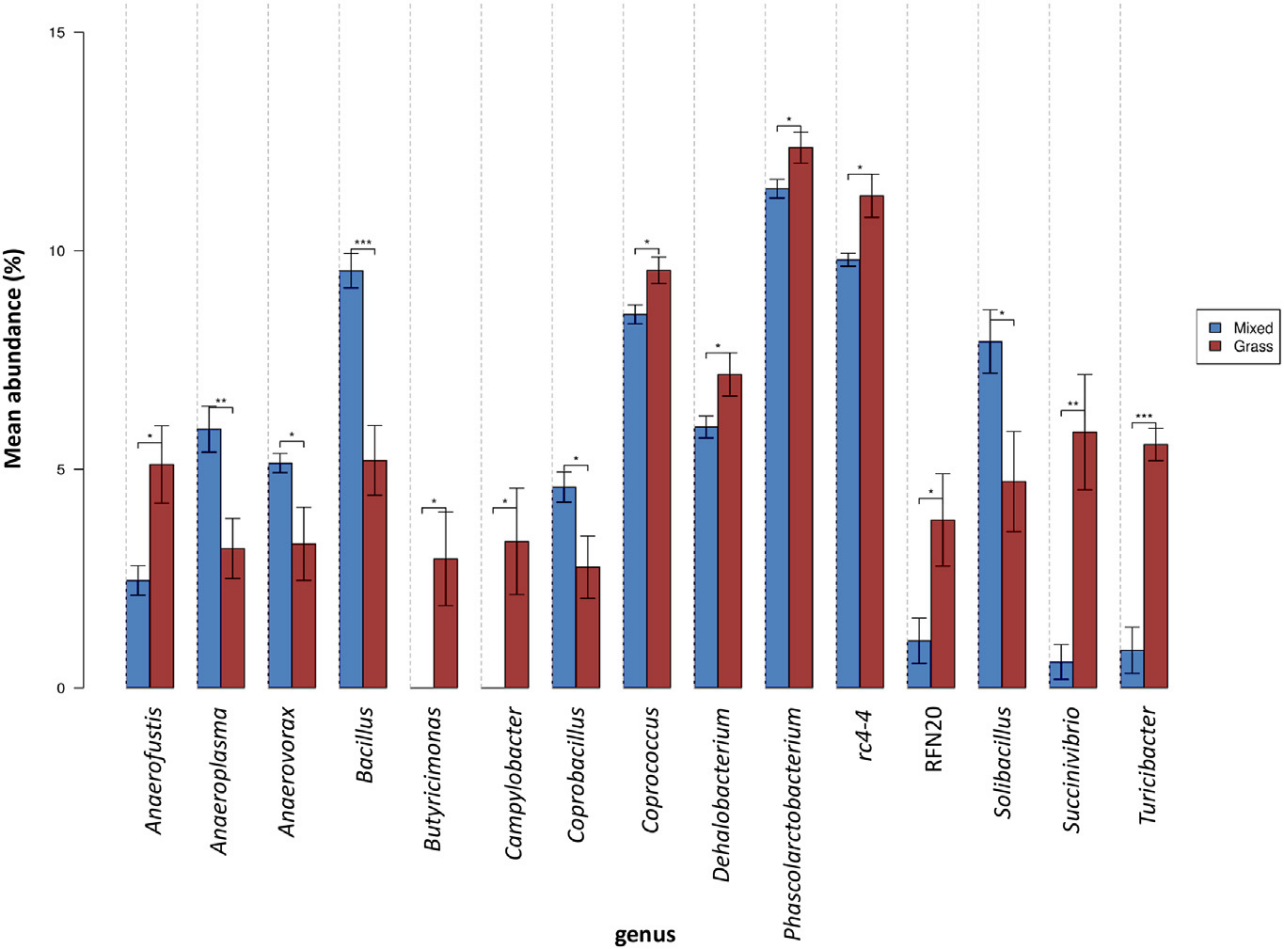
The effects of dietary differences on the community structure at genus level. Only taxa with significant difference are displayed and pair-wise comparisons were performed by Student's *t*-test (*p < 0.05; **p < 0.01; ***p < 0.001). Error bars represent standard error. Mixed, blue, Group M; grass, red, Group G.

Significant differences in abundance between the two dietary groups were observed for 15 bacterial populations at the genus level (Fig. 5). The relative abundance of *Anaeroplasma*, *Anaerovorax*, *Bacillus*, *Coprobacillus* and *Solibacillus* was significantly increased in Group M as compared to Group G. On the other hand, a significant reduction in abundance was observed for *Anaerofustis*, *Butyricimonas*, *Campylobacter*, *Coprococcus*, *Dehalobacterium*, *Phascolarctobacterium*, *rc4.4*, RFN20, *Succinivibrio* and *Turicibacter*. Overall, *Bacillus* had the most significant (p < 0.001) gain in abundance, while the abundance of *Turicibacter* decreased most significantly (p < 0.001) in Group M as compared to Group G. Furthermore, we detected that 369 (28.8%) of the 1282 observed OTUs unique to Group M were associated with these 15 genera which had significant differences in abundance among the two dietary groups. It was noted that the unique OTUs for *Anaerofustis*, *Butyricimonas* and *Campylobacter* were identified in Group G only.

## 4 Discussion

In the present study, we examined the potential effect of an environmentally-induced change of diet from herbivorous to omnivorous on the gut microbiota composition of cattle. As most of the feral cattle in Hong Kong roam freely in country parks and have access to a variety of food in addition to grass and vegetation, an herbivorous control group was not available locally. Instead, we sampled from cattle located on a free-grazing farm in Guangzhou, a city located just 119 km from Hong Kong, with a main diet of only grass and plants, which serves as an ideal herbivore gut microbiome control for comparison. Studies have also shown that while two cattle groups may be based in different geographical locations, when a comparable feed ration and management practice was used, the microbiome community between the two groups is observed to be highly similar in composition [17].

As our study examines free-living animals in their natural environment, there is no control over the type and quantity of food which is consumed, as opposed to animals housed under controlled experimental conditions which are provided with an artificial diet for a defined period of time. We have thus attempted to reduce complexity and minimize differences between our two sample groups by choosing cattle of the same breed and similar age as it has been observed that microbiota diversity and species richness is different between breeds [18] and from birth to adulthood [19]. The two cattle populations were also located in close proximity to each other with similar types of grassland and weather conditions and we have sampled in the same season as it is recognized that seasonal changes can affect grass availability and different feeds can alter the gut passage time of the cow [20]. Overall, our findings show a change in diet from 100% forage consumption to a mixed diet of forage and barbeque food waste resulted in a more diverse and rich microbiome, with nearly 1300 more OTUs identified on average (Fig. 3). In addition, results from PCA (Fig. 4) and ANOVA analysis (Fig. 5) revealed significant differences in bacterial community composition between the two microbiomes. The increase in microbiome diversity in the gut of the omnivorous cattle may be explained by their more diverse diet than that of the herbivorous cattle. This is in line with previous evidence that the diversity of a microbiota is enhanced as the diversity of the diet increases [21].

The observed changes in the gut microbiome composition may reflect the change in food digestion and energy production of the omnivorous cattle. Microbial populations in the rumen of the cow essentially act as a fermentation tank, digesting and fermenting food consumed by the animal, which provide nutrients and energy for the growth of the host [17,22]. On the other hand, these nutrients and breakdown products are also required by the microbes for their own survival as they thrive in the anaerobic environment of the rumen. A mutual relationship thus exists where the cow and microbes benefit and complement each other. This connection is primarily driven by diet as a change in food intake may result in the gain or loss of a particular substrate which is required for the survival of the microbe and provide competitive advantage of specific species over others. Changes in the abundance of certain bacteria in the omnivorous cattle may reflect the increased protein intake and reduced carbohydrate and fiber ingested by the animal. For example, a relatively higher abundance of *Bacteroides* and *Clostridium* has been observed in humans who consumed more protein and fat [23]. Similarly, the abundance of *Bacteroidaceae* and unclassified *Bacteroidales* are more enriched in the gut microbiota of the omnivorous cattle (Fig. 2B). The increase in abundance of the *Bacillus* genus in the omnivorous gut microbiota (Fig. 5) may also be related to the inclusion of meat in the diet. *Bacillus* is a genus of amylolytic, pectinolytic, lipolytic and cellulolytic bacteria that is also characterized by high proteolytic activity [24]. *Bacillus* proteolytic enzymes contribute to the normal digestion and degradation of protein, and produce amino acids that can act as major precursors for the synthesis of short-chain volatile fatty acids (SCFAs). SCFAs, typically acetate, propionate and butyrate, are an important source of energy for the animal and are produced primarily from bacterial fermentation of resistant starch, dietary fiber, and simple sugars, and to a lesser degree from unabsorbed or undigested proteins [25]. In the omnivorous cattle, a decrease in grass consumption due to the introduced intake of meat may result in reduced availability of SCFAs from the fermentation of dietary fiber and carbohydrate. An increase in *Bacillus* abundance may generate more SCFAs from proteins to maintain feed efficiency. Similarly, there was an increase in the abundance of *Anaerovorax* in the gut microbiota of the omnivorous cattle (Fig. 5). This genus is involved in the conversion of putrescine to butyrate [26] and may contribute to providing additional energy sources for the animal via the production of SCFAs. These findings suggest the adaptation of the gut microbiome of an herbivore to an increased protein diet and the potential importance of SCFA production in relation to such adaptation.

The reduced relative abundance of *Coprococcus*, *Butyricimonas*, *Succinivibrio*, *Campylobacter* and *Phascolarctobacterium* in the omnivorous gut microbiota (Fig. 5) likely reflects the reduced grass consumption as compared to Group G. *Coprococcus* and *Butyricimonas* are both butyrate-producing bacteria and utilize the fatty acids produced from carbohydrate fermentation to produce butyrate and propionate [27] or butyric and isobutyric acid [28], respectively. *Succinivibrio* ferments glucose to produce acetic and succinic acids, which aid in the metabolism of different types of fatty acids [29], while some isolates of *Campylobacter* have been reported to catabolize glucose [30,31]. *Succinivibrio* has also been observed to be more abundant in cattle on a high starch diet [10]. *Phascolarctobacterium*, on the other hand, is a propionate and acetate producer that utilizes succinic acid produced by other bacteria, such as *Succinivibrio*, for fermentation. We speculate that the decrease in grass intake by the omnivorous cattle may reduce the availability of carbohydrates, consequently resulting in a decrease in the relative abundance of bacteria genera that rely directly and indirectly on these substrates for their survival.

The changes in relative abundance of the six bacterial genera, *Turicibacter*, *rc4-4*, *Dehalobacterium*, *Coprobacillus*, RFN20 and *Anaerofustis* observed in this study (Fig. 5) are also in line with previous reports. Studies have reported positive association for the genera *Turicibacter* [32] and *rc4-4* [33] with a cellulose diet as well as negative association of *rc4-4* with a high fat, low dietary fiber diet [33]. The genus *Dehalobacterium* has been shown to be positively correlated with a glucose diet [34], while *Coprobacillus* have a negative correlation with glucose [35]. In addition, RFN20 is positively correlated with a high fiber diet [36] and *Anaerofustis* have been observed to be positively associated with the concentration of fecal dimethylamine, a choline metabolite which is reduced in the feces of rats on a high fat diet [37].

The increase in the relative abundance of *Anaeroplasma* (Fig. 5) may be related to the increased consumption of resistant starch, a type of starch that is not easily digested by cattle. Common food that are cooked at barbeque sites in Hong Kong include corn, bread, sweet potato, taro and yam which contain high levels of resistant starch. The consumption of these foods by the feral cattle may promote the growth of *Anaeroplasma*, and the amylolytic enzymes produced by this genus [38] may contribute to the breakdown of amylase, a form of resistant starch.

The rumen of cattle contains a rich and diverse collection of microbes that functions to enzymatically digest and ferment all materials ingested. This composition of the gut microbiome is tightly linked to the ability of the cow to extract energy from its feed, with SCFAs being an important component that serves the energy needs of the animal [17]. We proposed that a reduction in the intake of grass by the omnivorous cattle may reduce the amount of SCFAs generated. To compensate for this, we observed an increase in the abundance of *Bacillus* and *Anaerovorax*, which uses amino acids and putrescine, respectively, to produce SCFAs [25,26]. This is in line with findings where metabolic changes are usually accompanied by the occupation and dominance of a different species which use the same resources to counterbalance the overall availability of metabolites which are of high value to the animal [22]. Similarly, the reduced consumption of grass by the omnivorous cattle may also reduce the availability of carbohydrates. Starch and glucose are essential substrates for the growth of *Coprococcus*, *Butyricimonas*, *Succinivibrio*, *Campylobacter* and *Phascolarctobacterium*, which directly and indirectly relies on the bioavailability of carbohydrates for survival.

Evidently, a gut microbiota with high diversity is generally considered beneficial for the health of the host [25] and there is evidence that a boost in butyrate concentration is associated with an improvement of metabolic health [39]. On the other hand, a microbiome with lower diversity and richness has been associated with higher energy harvest from feed [22]. In our study, we observed that an inclusion of meat consumption and reduced grass intake resulted in a more diverse microbiome. The increased variation in feed most likely required the microbiome of the omnivorous cattle to adjust to a more complex bacterial composition that can specialize in different areas of fermentation to support the energy requirements of the host. In conclusion, we have observed that the rumen microbial ecosystem of the omnivorous cattle was significantly different to that of the herbivorous cattle. We speculate that the change of diet is one of the factors that resulted in a more diverse and rich microbiome to compensate for the potential reduction in energy productivity in order to improve feed efficiency and maintain the survival of the animal.

## Author Contributions

S.K.P.L. conceived of the study, designed the study and wrote the manuscript. J.L.L.T. performed the laboratory work, contributed to the interpretation of results and wrote the manuscript. T.H.C. carried out the bioinformatics and statistical analyses and wrote the manuscript. E.C. contributed to the interpretation of results and wrote the manuscript. A.K.L.T. carried out the bioinformatics and statistical analyses. G.P. gave advice on the bioinformatics analyses. S.L.Z. collected samples. P.C.Y.W. conceived of the study, designed the study, contributed reagents and revised the manuscript. All authors read and approved the manuscript.

## Funding

This work is partly supported by the Strategic Research Theme Fund, the Small Project Fund, The University of Hong Kong; and Croucher Senior Medical Research Fellowship, Croucher Foundation, Hong Kong. Moreover, this work is also partly supported by Guangdong Provincial Department of Science and Technology (Grant Nos. 2016A040403083 and 2016B020234006) and Guangdong Provincial Agricultural Department (Grant No. 2016LM3177).
